# Coproduction of Research Questions and Research Evidence in Public Health: The Study to Prevent Teen Drinking Parties

**DOI:** 10.1155/2017/3639596

**Published:** 2017-06-14

**Authors:** Mark Wolfson, Kimberly G. Wagoner, Scott D. Rhodes, Kathleen L. Egan, Michael Sparks, Dylan Ellerbee, Eunyoung Y. Song, Beata Debinski, Albert Terrillion, Judi Vining, Evelyn Yang

**Affiliations:** ^1^Department of Social Sciences and Health Policy, Wake Forest School of Medicine, Medical Center Boulevard, Winston-Salem, NC 27157, USA; ^2^Center for Research on Substance Use and Addiction, Wake Forest School of Medicine, Medical Center Boulevard, Winston-Salem, NC 27157, USA; ^3^SparksInitiatives, 1667-A South Kihei Road, Kihei, HI 96753, USA; ^4^Center for Injury Research and Policy, Johns Hopkins Bloomberg School of Public Health, 624 N. Broadway, Baltimore, MD 21205, USA; ^5^Community Anti-Drug Coalitions of America, 625 Slaters Lane, Suite 300, Alexandria, VA 22314, USA; ^6^Long Beach AWARE, 20 W Park Avenue, Suite 303, Long Beach, NY 11561, USA; ^7^Community Science, 438 N. Frederick Avenue, Suite 315, Gaithersburg, MD 20877, USA

## Abstract

Community-based participatory research (CBPR) provides a set of principles and practices intended to foster coproduction of knowledge. However, CBPR often has shortcomings when applied to population-level policy and practice interventions, including a focus on single communities and a lack of focus on policy change. At the same time, community trials focused on policy have shortcomings, including lack of stakeholder involvement in framing research questions and modest engagement in study implementation and interpretation and dissemination of results. We describe an attempt to hybridize CBPR and community trials by creating a partnership that included a national membership organization, a coalition advisory board, intervention and delayed intervention communities, and an academic study team, which collaborated on a study of community strategies to prevent underage drinking parties. We use qualitative and quantitative data to critically assess the partnership. Areas where the partnership was effective included (1) identifying a research question with high public health significance, (2) enhancing the intervention, and (3) improving research methods. Challenges included community coalition representatives' greater focus on their own communities rather than the production of broader scientific knowledge. This model can be applied in future attempts to narrow the gap between research, policy, and practice.

## 1. Introduction

Over the past twenty years, multiple fields—including medicine, social work, education, and public health—have come to recognize a disjuncture between research and practice. To take just one example, in the field of medicine, this disconnect has been famously described as the “17-year gap,” based on Weingarten and colleagues' [1] finding that “it takes 17 years to turn 14 percent of original research to the benefit of patient care.” Thus, the gap reflects both “attrition” of research findings (failure to translate into practice) and the length of time it takes for findings that do take root in practice to do so.

As in other fields in which the evidence-based practice “movement” has emerged [2, 3], policymakers, researchers, and practitioners in the substance abuse arena have discovered what many perceive to be a chasm between research and practice [4]. Researchers examining the relationship between “science” and “practice” often found practice to be wanting. For example, Ringwalt and colleagues [5] found that 82% of schools in a national sample of middle schools had adopted one or more substance abuse prevention curricula, but only 27% were using a curriculum that had been shown to be effective in prior research (also see Gottfredson and Wilson [6]). Nelson and colleagues [7] found relatively slow uptake by colleges and universities of research-based national recommendations for addressing high risk drinking by college students. Moreover, it was argued that even when evidence-based approaches had been adopted, they were not being implemented with fidelity to the model on which they were based [8].

However, this initial, rather one-sided discourse—which perhaps can be labeled “blame the practitioner”—was found to be too simplistic. Attention turned to the researcher, the dissemination process, and, finally, the research itself. For example, it was noted that most prevention research is focused not on interventions but on rates of behaviors of interest or correlates or antecedents of the behavior. It was also pointed out that much of the research identifying effective programs or initiatives had not been replicated [9]. Thus, a prevention program or approach, declared to be effective, might have only been subjected to one test—and that test was likely to have been in a highly controlled setting—making generalizability to other settings and nonresearch contexts unknown. Moreover, when researchers evaluated programs they had developed—and then tested as part of a trial—they were much more likely to find evidence of effectiveness than when a neutral third party conducted the evaluation [10]. And replications of interventions that had been found to be effective in controlled “efficacy” trials often showed no effects in “effectiveness” trials in “real world” settings [11]. Finally, a broader critique of expert knowledge argued that scientific expertise was being privileged, cutting against the grain of efforts to “democratize” clinical and societal decision-making by balancing the influence of experts with the input of ordinary citizens, including individuals directly affected by a condition or problem [12, 13].

Another criticism of translation of research to practice has to do with the ways in which evidence-based practices are promoted. For example, it is frequently argued that there is a lack of guidance on modification: how much alteration of a practice is acceptable in order to tailor it to the particular population being served [14]? There is sometimes resentment that innovations are externally “induced” by the requirements or preferences of funders. This may lead to unenthusiastic, or even ritualistic, implementation [15]. Finally, there are concerns about the ways in which practices get designated as evidence-based. Often funders of prevention efforts have developed official registries of “best,” “evidence-based,” or “promising” practices and incentivized the selection of approved practices for implementation (e.g., http://www.nrepp.samhsa.gov/). A few researchers have criticized the processes and criteria used for identifying programs as effective, arguing that there is considerable room for investigator bias and lack of rigor (e.g., Gorman [16]).

“Practice-based evidence” has come to be seen as an important antidote to the failures of evidence-based practice to come to full fruition [17]. In particular, community-based participatory research (CBPR) has been advocated as a research approach that may result in findings that are more likely to translate into widespread practice because of its focus on asking practice-relevant questions, participatory implementation processes, systems change, and widespread dissemination of findings [18].

More broadly, writers applying social, cultural, and historical perspectives on science have conceptualized the “coproduction” of knowledge, involving the participation of a variety of societal actors [19]. CBPR clearly serves as a prominent example of a set of principles and practices intended to foster coproduction of evidence and knowledge. CBPR has achieved considerable traction as an approach, as evidenced by government- and foundation-expressed interest in CBPR [20] and a proliferation of funded studies and publications that use a CBPR approach [21, 22].

That said, it is important to note that many CBPR projects focus on a single site, which may limit generalizability [23, 24]. Moreover, population-level policy and practice interventions, which often have more “reach” than typical individually or group-focused interventions, often benefit from, or require, inclusion of multiple communities. For example, community trials often include a number of design features that enhance internal, external, and statistical conclusion validity (such as multiple sites, randomization, and explicit accounting for nested data) (e.g., [25, 26]; see Boruch, [27] for an overview of methodological issues). These trials may provide solid designs for assessing the effects of interventions aimed at community change and may involve some level of community participation in designing and implementing interventions. However, such participation typically does not extend to the choice of the problem, study design and methods, and interpretation and dissemination of results. Moreover, as typically practiced, CBPR seldom involves pursuit of policy change—particularly changes in state or local public policy—as an intervention strategy [28]. And when CBPR is used to pursue policy change, the focus is typically a single community, limiting generalizability [28, 29].

This paper documents and critically analyzes a “macro” approach to CBPR, involving multiple communities, including structures that enable stakeholder and “community” input prior to the selection of study communities. In this paper, we describe the collaborative process that led to the development of this approach, as well as data on its functioning.

The overall study was a group-randomized multicommunity trial assessing the impact of a comprehensive community approach on preventing teen drinking parties. Twenty-four community coalitions from seven US states were recruited to be randomized to either an intervention or a delayed intervention condition. Over a 3-year period, intervention coalitions received training, technical assistance, and modest funding to catalyze and support efforts to plan and implement strategies related to policy, enforcement, and public education to respond to the problem of teen drinking parties in their communities.

The multilevel approach to CBPR we employed in the study is innovative in that it integrates features of randomized, multicommunity trials and CBPR principles and practices (building on the works of Cohen et al. [30] and Seifer et al. [31]). We aimed to engage national and community stakeholders in identifying and framing the research question, planning the proposed trial, obtaining funding, collaborating on implementation, and interpreting and disseminating results. Moreover, the approach was focused on changing local policy, which is rare in CBPR work [28].

## 2. Materials and Methods

The data presented in this paper reflect a case history approach to documenting and assessing the multilevel partnership for this study [32]. Data are from multiple sources, as outlined below.

### 2.1. Participant Observation

We participated in, and reviewed notes from, meetings, including meetings that led to the development of the focus on teen drinking parties and social host ordinances, meetings of a coalition advisory board (CAB) (described below) which were convened in order to partner with the Wake Forest School of Medicine (WFSM) study team on the development of funding proposals, and meetings of various groupings of the partners to carry out the collaborative project. Members of the WFSM study team also participated in visits to each of the 11 communities actively involved in carrying out the intervention. This involved meetings with coalition staff, community members (including youth and parents), law enforcement officials, and other local policymakers, including mayors, city council members, county commissioners, school superintendents, and city and county attorneys.

### 2.2. Community-Based Participatory Research Tracking Database

**Figure 1:**
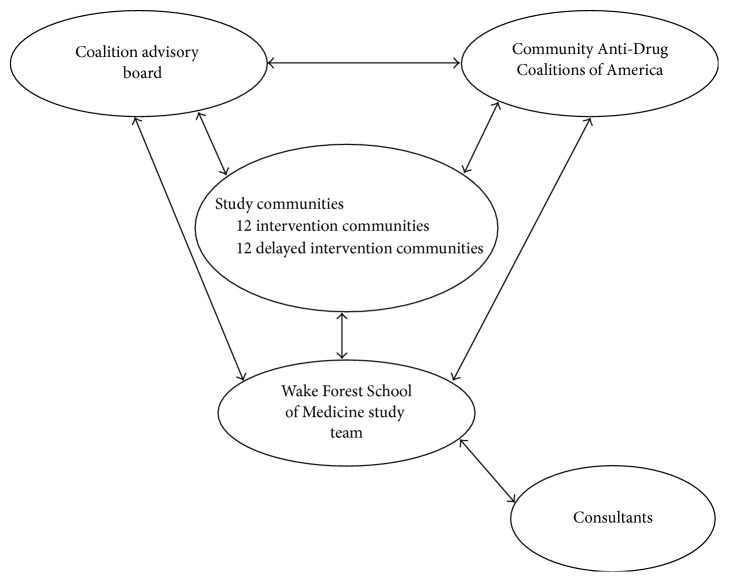
Organization of the community/research collaborative.

The WFSM study team maintained a CBPR tracking database, in which we systematically recorded contributions of the study team's partners in this effort, which included intervention sites, delayed intervention sites, the CAB, and Community Anti-Drug Coalitions of America (CADCA) (see Figure 1; this partnership is also described in greater detail below).

### 2.3. Ownership and Partnership Survey

We also draw on data from the Ownership and Partnership Survey, which was administered as a web survey in November 2015. The coordinators of each of the 11 coalitions that were implementing the intervention were invited to participate; 10 coordinators completed the survey (a response rate of 91%). In this paper, we report data from three constructs assessed on the survey: motivations for participation, benefits of participation, and drawbacks of participation.

The first construct, motives for participation, was measured using a set of items developed by the study team involving intervention site coalition coordinator perceptions of the importance of various potential motivations for involvement in the study including (1) direct benefit to one's community, (2) direct benefit to the intervention communities as a whole, (3) contributions to knowledge, and (4) being part of a national research study. The stem of this question was “this study is important to me because….” Seven potential motives, based on these four benefits, were presented as response options, shown in Table 3.

The second construct, benefits of participation, was assessed using an item adapted from Metzger et al. (2005). The stem of the item we used was “please indicate whether you and/or your coalition have experienced the following benefits as a result of your participation in the study” (the specific potential benefits are listed in Table 4).

The third construct, drawbacks of participation, was also assessed using an item adapted from Metzger et al. (2005). The stem of the item was “please indicate whether you and/or your coalition have experienced the following drawbacks as a result of your participation in the study” (the specific potential benefits are listed in Table 4).

### 2.4. Partnership Process Survey

We also draw on data from the Partnership Process Survey, which was conducted as a web survey, conducted in January 2016. We report data from two constructs on the survey, benefits and drawbacks of participation, which parallel the measures used to assess perceived benefits and drawbacks among the intervention coalition coordinators, described above.

All 8 CAB members who were involved in the study at the time of the survey completed it (100% response rate).

## 3. Results

### 3.1. Identification of the Topic, Development of the Collaborative, and Preparation and Submission of Grant Proposal

We used a multilevel approach to CBPR which was centered on partnerships with three groups: CADCA (a national membership organization representing community coalitions and other groups and individuals working to prevent alcohol, tobacco, and other drugs abuse at the community level), a coalition advisory board (CAB), and the 24 local coalitions which served as intervention or delayed intervention communities in the study.

The CAB grew out of CADCA's Community/Researcher Partnership Project. This initiative, which was supported by funding from the National Highway Traffic Safety Administration to CADCA, was designed to (1) help coalitions and researchers understand the research process, (2) partner coalitions with researchers through meetings and conferences about community issues, and (3) foster the identification of shared research questions and assist collaborative research projects. CADCA convened small meetings (15–30 individuals) of community coalition representatives, substance abuse researchers, and federal partners (see Table 1). The general problem of teen drinking parties, and a specific set of solutions involving community education, law enforcement, and policy development, was repeatedly prioritized by the participants at these meetings as one of the one or two top topics in need of research. They advocated for tests of the feasibility of implementing such changes in local communities and the effects of these changes on the size, frequency, and pervasiveness of underage drinking parties and attendant consequences. The specific policy change of interest was local “social host” ordinances, which hold the hosts of parties civilly or criminally responsible for underage drinking parties that take place in locations under their control, such as personal residences [33].

The WFSM study team had done conceptual and observational research focused on social host ordinances [33, 34]. We had also participated, and taken an active role in, the discussions about research priorities at the 2007 and 2009 CADCA-sponsored meetings of the Community/Researcher Partnership Project. Based on the identification of youth drinking parties and social host ordinances and related strategies at these two meetings and our long-standing interest in the process and impact of local social host initiatives, the study team joined forces with CADCA to identify and recruit a national advisory board of coalition leaders with experience in these issues in their communities. Most CAB members were recruited in late 2009 and early 2010 (see Table 1). The membership of the CAB varied between 8 and 10 members over its lifespan.

CADCA and the WFSM study team worked with the CAB to develop a proposal through a face-to-face meeting during the CADCA National Leadership Forum in February 2010, web-assisted conference calls in April and September 2010, and multiple telephone and email exchanges. In October 2010, an initial proposal was submitted to the National Institute on Alcohol Abuse and Alcoholism (NIAAA), which is a component of the US National Institutes of Health. That proposal fared well in the peer review process but was not scored at a level that resulted in funding based on the first submission. The collaboration of the WFSM study team, CADCA, and the CAB participated in a web-assisted conference call in May 2011 to plan revisions to the proposal, resulting in the submission of a revised proposal in October 2011. In April 2012, a 5-year research grant was funded by NIAAA (see Table 1). The study is using a CBPR approach to assess the process and impact of policy development, community education, and related enforcement efforts on preventing teen drinking parties.

### 3.2. Partner Involvement in Study Implementation

A summary of the ways in which community partners—the CAB, CADCA, intervention sites, and delayed intervention sites—participated in the study, using the dimensions of CBPR identified in AHRQ's analytic framework for community-based participatory research [35], is presented in Table 2. Specific examples of involvement and key contributions are presented below.

#### 3.2.1. Contributions of Partners in Design and Implementation of the Intervention

The greatest interest and involvement from members of the CAB were in design and implementation of the intervention. Some specific examples included a member of the CAB who was a retired police executive (Captain) who had previously worked closely with the coalition in the city he served. His roles in his own community included compiling data to make the case that a social host ordinance was needed, coordinating the coalition's advocacy efforts for the ordinance with the police department, ensuring that the ordinance, once passed, was enforced, and assessing the impact of the ordinance and related enforcement on calls for service to respond to complaints about loud and unruly parties. In his capacity as a member of the CAB, this individual worked with the study team to develop and implement trainings for intervention sites and directly participated in a number of on-site trainings, especially in the critical area of engaging law enforcement representatives in the intervention.

In addition, individuals from intervention sites who had expertise in a particular area of implementation participated in the delivery of trainings to the intervention sites as a whole. For example, a staff member of one of the intervention coalitions who had extensive experience in media advocacy and community education using social media played a key role in training intervention sites in the production and dissemination of social media messages about the moral and legal liability associated with hosting underage drinking parties.

#### 3.2.2. Contributions of Partners to Research Methods, Measurement, and Implementation

Members of the partnership also made important contributions to methods and measures. For example, one member of the CAB worked closely with the study team in the conceptualization and development of measures of implementation of the intervention—or “site-level dose”—which is a highly underdeveloped, but vitally important, aspect of the methodology of community prevention trials (see Wolfson et al. [26, 36]). The development of such measures enables assessment of (1) the degree of implementation and (2) whether there is a dose-response relationship between implementation and desired outcomes.

Second, multiple representatives of the CAB and intervention site coalitions reviewed and provided input on drafts of the web-based Millennial Youth and Young Adult Survey (“MYSurvey”), which was developed by the study in order to assess key outcomes. In order to boost response rates following the first fielding of the MYSurvey, we convened a work group of CAB members and representatives from intervention coalitions to generate and evaluate ideas about changes that we could make to the survey design and fielding. The work group met once by a web-assisted call where it decided on some initial modifications for the second fielding, and subsequent decisions were made through email discussion about remaining options. Further feedback and suggestions were sought at the second in-person intervention training.

Several representatives from delayed intervention coalitions also helped to generate ideas and make recommendations for changes to the survey over a web-assisted call. Finally, in response to a decision made by the work group and endorsed by other coalition representatives, the study team revised survey mailing materials to include photographs of iconic features of their communities, so that we could tailor the look of survey materials for each community. To achieve this, the study team enlisted both intervention and delayed intervention coalitions to help identify which features would be appropriate and to provide photographs of well-known community landmarks. These and other changes that were made to the survey following the first fielding contributed to an almost 50% increase in responses between the first fielding and the fourth fielding of the survey.

In addition, a focus group of law enforcement representatives from the intervention coalitions was assembled at the second in-person intervention training also to review the law enforcement survey, which measured key implementation factors such as departmental activities around underage drinking parties. The group generated and discussed ideas about changes that could be made, and these were incorporated into subsequent survey fieldings. Again, we observed about a 50% increase in responses.

#### 3.2.3. Contributions of Partners to Interpretation and Dissemination of Results

After each fielding of the MYSurvey, we created and distributed a report highlighting key data for both intervention and delayed intervention coalitions. This includes personalized (community-specific) data for each community, so coalition members can evaluate how their community is doing relative to the other communities in the study (in the aggregate), with respect to problems of youth alcohol use and underage drinking parties. These reports were also a useful vehicle for discussing data with the community coalitions. For example, they enabled the study team to have useful discussions with intervention coalition leaders on how selected data could be used to make the case to key stakeholders, including policymakers, and on the importance of enforcement and policy development efforts to address teen drinking parties.

In addition, an interpretation of results meeting took place in February 2017, in conjunction with CADCA's annual National Leadership Forum outside of Washington, DC. Members of the CAB, CADCA representatives, and both intervention and delayed intervention coalitions participated in this meeting. The meeting served as a vehicle for (1) sharing preliminary results (with respect to both implementation and outcomes), (2) systematically soliciting input on how the results should be interpreted, (3) obtaining feedback on the technical assistance, training, and other supports provided by the study team, CADCA, and the CAB to the intervention sites for planning and implementing the intervention (technical assistance and training), and (4) eliciting ideas about the most effective ways of disseminating study findings for a variety of policymaking, practitioner, and scientific audiences.

### 3.3. Community Coalition Leader Motives for Involvement

Intervention site coalition leaders' perceptions of the importance of various motives for participation in the study are presented in Table 3 (based on data from the 2015 Ownership and Partnership Survey). All of the motives were endorsed (either “strongly agree” or “agree”) by the majority of the intervention site coalition coordinators; consequently, we use “strongly agree” versus all other responses (“agree,” “neither agree nor disagree,” “disagree,” and “strongly disagree”) as the contrast of primary interest. A number of important patterns are observed in these data. First, local coalition leaders—perhaps not surprisingly—are most likely to strongly endorse what might be called (nonpejoratively) “parochial” motives—including direct benefit to one's community with respect to development and implementation of effective strategies (strongly agreed to by nine out of ten respondents) and learning whether a comprehensive approach to the problem is effective in one's own community (strongly agreed to by eight out of ten respondents). The parallel items for all of the participating intervention communities were strongly endorsed by far fewer of the respondents (four out of ten strongly agreed with wanting all of the intervention communities to develop and implement effective strategies, and the same number and proportion strongly agreed to learning whether the approach was effective in all of the intervention communities). Interestingly, a somewhat larger number—seven out of ten—strongly agreed that the study was important to them because it will make an important contribution to knowledge on the impact of such approaches more generally (i.e., not limited to the intervention communities participating in this study). Half of the respondents indicated that being part of a national research study was important to her or him, and half indicated it was important for their community.

Table 4 presents the results of survey questions posed to both CAB members and intervention coalition representatives about the benefits and drawbacks of participation in the study. These two groups had strikingly similar views of the benefits of participation, with large percentages endorsing benefits related to their ability to address an important issue, acquisition of useful knowledge, and enhanced ability to affect public policy. There was one benefit, however, that showed a dramatic difference between these two groups: 75% of CAB members, but only 40% of intervention site representatives, believed that participation in the study would help them acquire additional financial support in the future. One CAB member expressed the following: “I had no expectation of [participation] impacting our funding…except maybe indirectly, in that the knowledge gained and the experience might make me better able to write more successful grants.”

There are striking differences in the way the two groups responded to the questions about drawbacks. Few CAB members endorsed any of the drawbacks. However, 90% of intervention site representatives indicated that they at some point experienced frustration or aggravation as a result of participation in the study, 40% indicated that there was a conflict between their job and the project work, 40% indicated that there was a diversion of time and resources away from other priorities or obligations, and 30% said that they were viewed negatively as a result of their association with the project. Based on our conversations and interviews with intervention coordinators, we believe that the perceived drawbacks are primarily associated with the substantial demands of policy advocacy—as each of the sites chose to pursue a social host ordinance, and, in cases where it was enacted, advocate for its vigorous enforcement.

## 4. Discussion

In this paper, we have examined the conceptualization, development, and implementation of a multilevel approach to CBPR, which integrated CBPR principles and practices and features of randomized, multicommunity trials. This was an attempt to address some of the shortcomings of conventional CBPR approaches such as the focus on a single community and the lack of focus on policy change. At the same time, it was an attempt to address some of the shortcomings of community trials focused on policy, such as lack of stakeholder involvement in framing the research question and moderate to minimal engagement of stakeholders in implementing the project and in interpreting and disseminating results.

We sought to accomplish this hybridization of CBPR and community trials—in large part—by creating a partnership structure that involved a national membership organization (CADCA), a coalition advisory board, intervention communities, delayed intervention communities, and the academic study team. In Results above, we note a number of areas where this partnership structure was beneficial. These include (1) identifying a research question with high public health significance, (2) enhancing the quality of the intervention, and (3) improving research methods and implementation (e.g., improving response rates).

The data we present also point to some challenges experienced with this approach. For example, CBPR projects—including ours—often have lofty goals that community members will become deeply engaged in interpreting results and in helping identify their importance for science and/or practice. Yet we found that the local coalition leaders with whom we worked were—understandably—more concerned with achieving outcomes and answering questions related to their own communities rather than the overall set of intervention communities. In addition, it has been more difficult for us to engage delayed intervention communities than intervention communities—in meaningful ways. And the coordinators of the intervention coalitions noted some important drawbacks, in addition to benefits, to participation in the study.

Coproduction of knowledge happened in multiple ways, as noted above. But the meetings within communities—involving individual intervention coalition staff, local policymakers and community members, CAB members (in some instances), and study team staff—produced some of the most important knowledge about barriers and facilitators to passage and enforcement of social host ordinances. In particular, these meetings reinforced the importance of local legal culture, such as the preference of many city attorneys for criminal ordinances, as opposed to civil ordinances, despite some evidence that civil ordinances are more efficacious [37]. In addition, most CAB member communities that had passed social host ordinances had criminal ordinances rather than civil ones. These discussions in the partnership for the study reinforced the common trade-offs between an academically defined best practice and practices that may be less effective, but more feasible to institutionalize, in local communities.

## 5. Conclusions 

In the current public health research environment, there is a dual emphasis, from funders and other sources, on rigorous trials that involve multiple sites, along with genuine engagement with a variety of stakeholders, including policymakers, community members and institutions, patients, and their families [38, 39]. This dual emphasis underscores the continuing, and likely growing, importance of developing effective approaches to engaging community coalitions, policymakers, and other stakeholders in both identifying key researcher questions that will inform policy and practice and carrying out rigorous studies to investigate these questions. The model presented in this paper could be incorporated into structural approaches to closing the gap between research, policy, and practice [40].

That said, our case study raises some important questions. First, what motivates organizations, such as community prevention coalitions, to participate in research studies? While there has been a fluorescence of research on individual participation (e.g., Williams et al. [41]; Hallowell et al. [42]), research on motivations for the participation of organizations is rare. We suggest that such research may be usefully informed by research on incentives both for organizations and for individuals to participate in the creation of public goods (e.g., Prestby et al. [43]).

A second critical question is how apparent contradictions in carrying out CBPR and randomized community trials can be reconciled. We employed a particular set of strategies to attempt to reconcile the two approaches. Perhaps the most important strategic choice was to split the “community” responsibilities and inputs into three fairly distinct groups. The first was CADCA—and the coalitions it brought together with researchers in early meetings—which enabled identification of a key research question with both scientific and practical public health significance (the feasibility and effectiveness of a set of strategies to prevent teen drinking parties). The second was the CAB—which worked closely with WFSM and CADCA to design the study and seek funding and provide advice on recruitment of study coalitions and the design of the intervention. The third group was the coalitions that were ultimately selected to participate in the study, which had the major responsibility for implementation of the intervention. While this approach in many respects worked well, there are important questions about its generalizability and replicability. And some advocates of CBPR may question the allocation of responsibilities for “community” input to any groups other than the communities ultimately responsible for implementing the intervention.

Other groups have begun to report some successes with similar approaches, such as the “community-partnered participation approach” reported by Stockdale and colleagues, which was built on long-standing relationships between academic and community partners [44]. In addition, there has been at least one other deliberate effort to “hybridize” CBPR and randomized community trials in order to promote translation and uptake of study findings [24]. These efforts—like ours—had to confront issues of potential trade-offs between participation and rigor and varying definitions of what constitutes “communities” and what constitutes “engagement.” It is clear that the feasibility, generalizability, and effectiveness of hybrid approaches to community trials and CBPR are a critical topic for future research on the coproduction of knowledge in public health.

## Figures and Tables

**Table 1:** Development of the collaborative and study.

Stage of development	Timeline of key activities
(I) CADCA partnership and CAB develop.	*(i) 2007, 2009*: CADCA-sponsored meeting in Alexandria and Louisville *(ii) Late 2009-Early 2010*: recruitment of coalition advisory board (CAB) members

(II) Development and 1st submission of grant proposal to NIAAA	*(i) February 2010*: social host CAB meeting during CADCA NLF (WFSM, CAB, CADCA) *(ii) April 2010*: web-assisted conference call (WFSM, CAB, CADCA) *(iii) September 2010*: web-assisted conference call (WFSM, CAB, CADCA) *(iv) October 2010*: first submission of SHO proposal to NIAAA; proposal received a very favorable review but was not funded on the 1st submission

(III) Revision and 2nd submission of grant proposal to NIAAA	*(i) May 2011*: Web-assisted conference call (WFSM, CAB, CADCA) *(ii) July 2011*: 2nd submission of SHO proposal to NIAAA *(iii) October 2011*: proposal received a favorable and fundable score *(iv) April 2012*: Grant awarded

**Table 2:** Community partner roles and responsibilities.

Research component	Community partner roles and responsibilities	Partners involved in process
Study designed and funding sought	Involved in designing study, refining study questions, and writing proposal.	Wake Forest study team CADCA Coalition advisory board

Participants recruited and retention systems implemented	Input on recruitment and retention strategies for communities, schools, and study participants. Incentives at each level.	Wake Forest study team CADCA Coalition advisory board

Measurement instruments designed and data collected	Input on relevant and appropriate measures for the youth, parent, law enforcement agency and coalition surveys and community data.	Wake Forest study team CADCA Coalition advisory board Intervention sites Delayed intervention sites

Intervention designed and implemented	Input on timing and topics in intervention trainings. Will be resource to communities for assessing social availability of alcohol and passing SHO policies.	Wake Forest study team CADCA Coalition advisory board Intervention sites

Data analyzed, interpreted, disseminated, and translated	Input on meaning and interpretation of the results. Participation in formulation of products and modes of delivery for disseminating results.	Wake Forest study team CADCA Coalition advisory board Intervention sites Delayed intervention sites

**Table 3:** Intervention site ratings of importance of reasons for participation in the study, 2015 Ownership and Partnership Survey (*n* = 10).

“This study is important to me because…”	Strongly agree (%)	Agree (%)	Neither agree nor disagree (%)	Disagree (%)	Strongly disagree (%)
I want to develop and implement effective strategies to prevent underage drinking parties in my community.	90	10	0	0	0

I want all of the intervention communities in the study to develop and implement effective strategies to prevent underage drinking parties.	40	40	20	0	0

We will learn whether a comprehensive approach to addressing underage drinking parties…is effective in my community.	80	20	0	0	0

We will learn whether a comprehensive approach to addressing underage drinking parties…is effective in all of the intervention communities.	40	50	10	0	0

It will make an important contribution to knowledge on the impact of comprehensive approaches to addressing underage drinking parties.	70	30	0	0	0

Being part of a national research study is important to me.	50	30	20	0	0

Being part of a national research study is important to my community.	50	30	10	0	10

**Table 4:** Coalition advisory board members (*n* = 8) and intervention coalition members (*n* = 10) perceptions of benefits and drawbacks experienced as a result of participating in the research study.

	Coalition advisory board (%)	Intervention sites (%)
*Benefits*		
Enhanced ability to address an important issue	100	100
Enhanced ability to work with local communities/your local community	87.50	90
Enhanced ability to work with researchers	100	90
Heightened public profile	100	90
Increased utilization of my expertise or services	87.5	100
Acquisition of useful knowledge about services, programs, or people in the community	87.5	100
Enhanced ability to affect public policy	87.5	100
Development of valuable relationships	100	90
Enhanced ability to meet the needs of my constituency or clients	87.5	80
Ability to have a greater impact than I could have on my own	100	100
Ability to make a contribution to the community	100	100
Acquisition of additional financial support (i.e., grant funds)	75	40
Training opportunities	87.5	100
*Drawbacks*		
Diversion of time and resources away from other priorities or obligations	25	40
Insufficient influence in study activities	12.5	0
Viewed negatively due to my association with the project	0	30
Frustration or aggravation	0	90
Insufficient credit given to me for contributing to the accomplishments of the project	0	10
Conflict between my job and the project work	0	40

## References

[1] Weingarten S., Garb C. T., Blumenthal D., Boren S. A., Brown G. D. (2000). Improving preventive care by prompting physicians. *Archives of Internal Medicine*.

[2] Hammersley M. (2005). Is the evidence-based practice movement doing more good than harm? reflections on iain chalmers' case for research-based policy making and practice. *Evidence and Policy*.

[3] Biglan A., Ogden T. (2008). The evolution of evidence-based practices. *European Journal of Behavior Analysis*.

[4] Glasgow R. E., Green L. W., Taylor M. V., Stange K. C. (2012). An evidence integration triangle for aligning science with policy and practice. *American Journal of Preventive Medicine*.

[5] Ringwalt C. L., Ennett S., Vincus A., Thorne J., Rohrbach L. A., Simons-Rudolph A. (2002). The prevalence of effective substance use prevention curricula in U.S. middle schools. *Prevention Science*.

[6] Gottfredson D. C., Wilson D. B. (2003). Characteristics of effective school-based substance abuse prevention. *Prevention Science*.

[7] Nelson T. F., Toomey T. L., Lenk K. M., Erickson D. J., Winters K. C. (2010). Implementation of NIAAA college drinking task force recommendations: how are colleges doing 6 years later?. *Alcoholism: Clinical and Experimental Research*.

[8] Dusenbury L., Brannigan R., Falco M., Hansen W. B. (2003). A review of research on fidelity of implementation: implications for drug abuse prevention in school settings. *Health Education Research*.

[9] Valentine J. C., Biglan A., Boruch R. F. (2011). Replication in Prevention Science. *Prevention Science*.

[10] Eisner M. (2009). No effects in independent prevention trials: can we reject the cynical view?. *Journal of Experimental Criminology*.

[11] Hallfors D., Cho H., Sanchez V., Khatapoush S., Hyung M. K., Bauer D. (2006). Efficacy vs effectiveness trial results of an indicated ‘model’ substance abuse program: implications for public health. *American Journal of Public Health*.

[12] Epstein S. (1998). *Impure Science: AIDS, Activism, and the Politics of Knowledge*.

[13] Hess D. J., Zavestoski P. B. S. (2005). Medical Modernisation, Scientific Research Fields and The Epistemic Politics Of Health Social Movements. *Social Movements in Health*.

[14] Lundgren L., Amodeo M., Cohen A., Chassler D., Horowitz A. (2011). Modifications of evidence-based practices in community-based addiction treatment organizations: a qualitative research study. *Addictive Behaviors*.

[15] Marcus A. A. (1988). Implementing externally induced innovations: a comparison of rule-bound and autonomous approaches. *Academy of Management Journal*.

[16] Gorman D. M. (2002). Defining and operationalizing ‘research-based’ prevention: a critique (with case studies) of the US Department of Education's Safe, Disciplined and Drug-Free Schools Exemplary Programs. *Evaluation & Program Planning*.

[17] Green L. W. (2008). Making research relevant: if it is an evidence-based practice, where's the practice-based evidence?. *Family Practice*.

[18] Israel B. A., Schulz A. J., Parker E. A., Becker A. B., Allen A., Guzman J. R., Minkler M., Wallerstein N. (2003). Critical issues in developing and following community-based participatory research principles. *Community-Based Participatory Research for Health*.

[19] Nowotny H., Scott P., Gibbons M. (2001). *Rethinking Science*.

[20] Minkler M., Blackwell A. G., Thompson M., Tamir H. (2003). Community-based participatory research: implications for public health funding. *American Journal of Public Health*.

[21] Kraemer Diaz A. E., Spears Johnson C. R., Arcury T. A. (2013). Variation in the interpretation of scientific integrity in community-based participatory health research. *Social Science and Medicine*.

[22] Wolfson M., Parries M., Jane B.-H., Sandra L., Mayer Z. (2010). The institutionalization of community action in public health. *Social Movements and the Transformation of American Health Care*.

[23] Faridi Z., Grunbaum J. A., Gray B. S., Franks A., Simoes E. (2007). Community-based participatory research: necessary next steps. *Preventing Chronic Disease*.

[24] Katz D. L., Murimi M., Gonzalez A., Njike V., Green L. W. (2011). From controlled trial to community adoption: the multisite translational community trial. *American Journal of Public Health*.

[25] Wagenaar A. C., Murray D. M., Gehan J. P. (2000). Communities mobilizing for change on alcohol: outcomes from a randomized community trial. *Journal of Studies on Alcohol*.

[26] Wolfson M., Champion H., McCoy T. P. (2012). Impact of a randomized campus/community trial to prevent high-risk drinking among college students. *Alcoholism: Clinical and Experimental Research*.

[27] Boruch R. F. (2005). Place randomized trials: experimental tests of public policy. *Annals of the American Academy of Political and Social Sciences*.

[28] Freudenberg N., Tsui E. (2014). Evidence, power, and policy change in community-based participatory research. *American Journal of Public Health*.

[29] Gonzalez P. A., Minkler M., Garcia A. P. (2011). Community-based participatory research and policy advocacy to reduce diesel exposure in West Oakland, California. *American Journal of Public Health*.

[30] Cohen D. A., Han B., Derose K. P., Williamson S., Marsh T., McKenzie T. L. (2013). Physical activity in parks: a randomized controlled trial using community engagement. *American Journal of Preventive Medicine*.

[31] Seifer S. D., Michaels M., Collins S. (2010). Applying community-based participatory research principles and approaches in clinical trials: forging a new model for cancer clinical research. *Progress in Community Health Partnerships*.

[32] Yin R. K. (2014). *Case Study Research: Design and Methods*.

[33] Wagoner K. G., Francisco V. T., Sparks M., Wyrick D., Nichols T., Wolfson M. (2012). A review of social host policies focused on underage drinking parties: suggestions for future research. *Journal of Drug Education*.

[34] Wagoner K. G., Sparks M., Francisco V. T., Wyrick D., Nichols T., Wolfson M. (2013). Social host policies and underage drinking parties. *Substance Use and Misuse*.

[35] Viswanathan M., Ammerman A., Eng E. (2004). Community-based participatory research: assessing the evidence. *Summary, Evidence Report/Technology Assessment*.

[36] Wolfson M., Champion H., Rogers T. (2011). Evaluation of free to grow: head start partnerships to promote substance-free communities. *Evaluation Review*.

[37] Paschall M. J., Lipperman-Kreda S., Grube J. W. (2014). Relationships between social host laws and underage drinking: findings from a study of 50 California cities. *Journal of Studies on Alcohol and Drugs*.

[38] Fleurence R., Selby J. V., Odom-Walker K. (2013). How the patient-centered outcomes research institute is engaging patients and others in shaping its research agenda. *Health Affairs*.

[39] Michener L., Cook J., Ahmed S. M., Yonas M. A., Coyne-Beasley T., Aguilar-Gaxiola S. (2012). Aligning the goals of community-engaged research: why and how academic health centers can successfully engage with communities to improve health. *Academic Medicine*.

[40] Molleman G., Fransen G. (2012). Academic collaborative centres for health promotion in the netherlands: building bridges between research, policy and practice. *Family Practice*.

[41] Williams B., Entwistle V., Haddow G., Wells M. (2008). Promoting research participation: why not advertise altruism?. *Social Science and Medicine*.

[42] Hallowell N., Cooke S., Crawford G., Lucassen A., Parker M., Snowdon C. (2010). An investigation of patients' motivations for their participation in genetics-related research. *Journal of Medical Ethics*.

[43] Prestby J. E., Wandersman A., Florin P., Rich R., Chavis D. (1990). Benefits, costs, incentive management and participation in voluntary organizations: a means to understanding and promoting empowerment. *American Journal of Community Psychology*.

[44] Stockdale S. E., Tang L., Pudilo E. (2016). Sampling and Recruiting Community-Based Programs Using Community-Partnered Participation Research. *Health Promotion Practice*.

